# Supplementary material for: Single Cell Transcriptomic and Chromatin Profiles Suggest Layer Vb Is the Only Layer With Shared Excitatory Cell Types in the Medial and Lateral Entorhinal Cortex
Source: Front Neural Circuits. 2022 Jan 26;15:806154. doi: 10.3389/fncir.2021.806154 (PMC8826650; doi:10.3389/fncir.2021.806154)
Supplement: Supplementary file 14 [file Data_Sheet_1.ZIP › Exc_vs_Inh_Motifs/homerResults/motif25.similar.html]

motif25

## Information for motif25

A
G
T
C
A
C
G
T
C
G
T
A
A
C
T
G
A
T
G
C
C
G
T
A
A
G
C
T
A
T
C
G
  
Reverse Opposite:  

A
T
G
C
C
T
G
A
A
C
G
T
A
T
C
G
A
G
T
C
A
C
G
T
C
G
T
A
A
C
T
G
  

|  |  |
| --- | --- |
| p-value: | 1e-81 |
| log p-value: | -1.887e+02 |
| Information Content per bp: | 1.912 |
| Number of Target Sequences with motif | 2303.0 |
| Percentage of Target Sequences with motif | 5.46% |
| Number of Background Sequences with motif | 390.9 |
| Percentage of Background Sequences with motif | 3.60% |
| Average Position of motif in Targets | 347.4 +/- 255.2bp |
| Average Position of motif in Background | 307.5 +/- 236.1bp |
| Strand Bias (log2 ratio + to - strand density) | -0.0 |
| Multiplicity (# of sites on avg that occur together) | 1.04 |
| Motif File: | file (matrix) reverse opposite |

### Similar de novo motifs found

|  |  |  |  |  |  |  |  |
| --- | --- | --- | --- | --- | --- | --- | --- |
| Rank | Match Score | Redundant Motif | P-value | log P-value | % of Targets | % of Background | Motif file |
| 1 | 0.632 | A G T C A C G T C G T A A C T G A G T C C G T A C G T A A G T C C G T A A C T G | 1e-79 | -182.975656 | 0.39% | 0.06% | motif file (matrix) |
